# Leiomyosarcoma of the Maxilla: A Case Report and Literature Review

**DOI:** 10.30476/DENTJODS.2021.89153.1398

**Published:** 2022-06

**Authors:** Mohammad Jafarian, Shahabaldin Azizi

**Affiliations:** 1 Dept. of Oral and Maxillofacial Surgery, Head of Dental Research Center, Research Institute of Dental Sciences, Shahid Beheshti University of Medical Sciences, Tehran, Iran; 2 Dept. Oral and Maxillofacial Surgery Dental School, Iran University of Medical Sciences, Tehran, Iran

**Keywords:** Maxillary tumor, leiomyosarcoma, Malignancy

## Abstract

Leiomyosarcoma is a malignant tumor arising from smooth muscle cells accounting for 10-20% of soft tissue sarcomas and less than 2% are located in head and neck region. We report a case of leiomyosarcoma of maxilla in a 26-year-old female patient referred to Shahid-Beheshti University of Medical Sciences, Faculty of Dentistry. Patient complained of swelling of left side of the face from 5 months ago without a history of significant illness. Clinical examination showed asymmetric swelling of left side of the face with tenderness. The lesion in the left buccal vestibule was tender to the touch and contained erythematous mucosa with a firm tissue. Results of the neck computed tomography (CT) scan indicated mucosal thickening in the left maxillary sinus. In the preoperative magnetic resonance imaging (MRI), a mass with an estimated size of 4.7×3.1×3.0cm was found, extending from the posterior wall to the posterolateral wall of the left maxillary sinus, causing impression and remodeling of the sinus wall. Incisional biopsy was preformed and immunohistochemistry suggested leiomyosarcoma. Partial maxillectomy of the posterior maxillary walls was performed, using an intraoral incision and dissection from the maxillary vestibule up to the mandibular vestibule. The tumor was excised using frozen section. Pathological examination reported the lesion compatible with leiomyosarcoma, with all surgical margins free of tumor. Post-operative MRI showed no definite mass in the region. In conclusion, according to the present results, the clinical features of leiomyosarcoma of the maxilla are clear, and diagnosis is feasible.

## Introduction

Leiomyosarcoma, which was first reported by Zieler [ 1 ], is a malignant tumor, arising from smooth muscles. According to statistics, it accounts for 10-20% of soft tissue sarcomas [ 2 ]. On the other hand, soft tissue sarcomas of the head and neck are rare, accounting for less than 10% of soft tissue sarcomas. Leiomyosarcoma is responsible for 4% of head and neck sarcomas [ 3 ]. However, only 1.1% of leiomyosarcoma cases are located in the head and neck regions [ 4 ], making it a rare type of tumor. Herein, we report a case of leiomyosarcoma of the maxilla in a 26-year-old woman, who was diagnosed and managed at Shahid Beheshti Faculty of Dentistry, Tehran, Iran. A written consent was obtained from patient for this case report, and all images were deidentified.

## Case Presentation

A 26-year-old female patient was admitted to the Department of oral and maxillofacial surgery, Shahid Beheshti University of Medical Sciences, Tehran, Iran. Swelling of the left side of the face was the chief complaint. She had noticed the lesion five months ago and visited a general dentist. Diagnosis of infection was made, and antibiotic therapy was initiated. She had a history of hospitalization due to nasal polyp surgery in the past year, in her past medical history with no history of smoking or alcohol/drug abuse or any significant illness. Her father had died due to lung cancer at the age of 52. 

### Clinical exam

**Figure 1:**
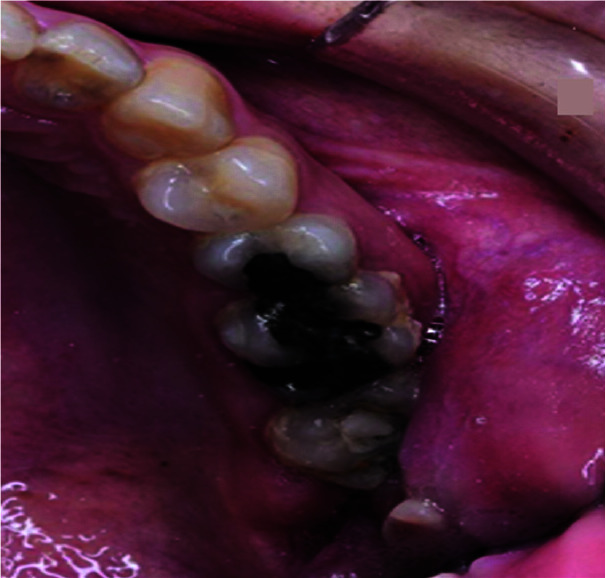
The lesion’s oral presentation

In oral and maxillofacial examination, asymmetry of the face with swelling on the left cheek was observable. The skin of the face and neck was normal. The area of swelling was tender, without paresthesia and or pain. The lesion in the left buccal vestibule was tender to the touch and contained erythematous mucosa with a firm tissue ( Figure 1). No other significant finding was observed in her complete exam.

### Paraclinical exams

**Figure 2:**
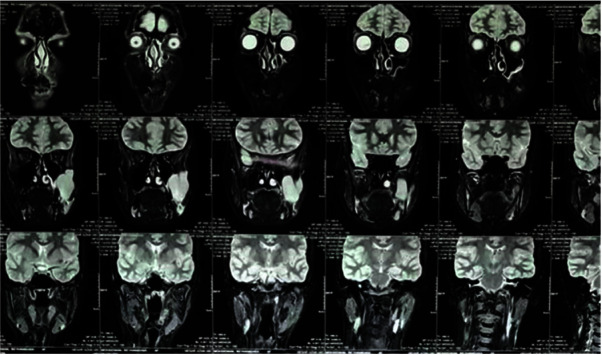
Preoperative MRI images

The complete blood cell count (CBC) and biochemical markers, including blood urea nitrogen (BUN), create nine, calcium, phosphorous, sodium, potassium, bilirubin (direct and total bilirubin), alanine aminotransferase (ALT), aspartate aminotransferase (AST), lactate dehydrogenase (LDH), alkaline phosphatase, urea, and vitamin D, were normal. The level of cancer antigen 125 (CA-125) tumor marker was also 7.6 (normal range &lt;35). A computed tomography (CT) scan was performed on the neck, thorax, pelvis, and abdomen. The results showed no abnormal features in the abdominal, thoracic, or pelvic areas. However, the results of the neck CT scan indicated mucosal thickening in the left maxillary sinus. In the preoperative magnetic resonance imaging (MRI), a mass with an estimated size of 4.7× 3.1×3.0cm was found, extending from the posterior wall to the posterolateral wall of the left maxillary sinus, causing impression and remodeling of the sinus wall. High signal changes were observed on the T2-weighted image, whereas low signal changes were observed on the T1-weighted image. These signal changes were attributed to the surrounding fat, which could be indicative of lymphadenopathy. Mild mucosal thickening was also seen in the left maxillary sinus ( Figure 2).

Because of the non-specific clinical presentations, an incisional biopsy was performed under local anesthesia. The gross pathology of the specimen consisted of an irregular, delicate, and creamy-brown tissue. The size of the specimen was 1.8×1.6×0.4 cm. The pathologic finding was a malignant neoplasm, composed of sheets of ovoid to spindle cells, with pleomorphism, hyperchromatism, high nucleus-to-cell ratio and mitosis. The lesion was covered by non-keratinized stratified squamous epithelium with exocytosis. The tumor cells had invaded muscle fibers. Accordingly, a provisional diagnosis of malignant spindle cell tumor was established. Generally, the differential diagnoses of spindle-shaped lesions include PUS, rhabdomyosarcoma (RMS), LMS, malignant peripheral nerve sheath tumor (MPNST) and fibrosarcoma [ 5 ]. 

**Figure 3:**
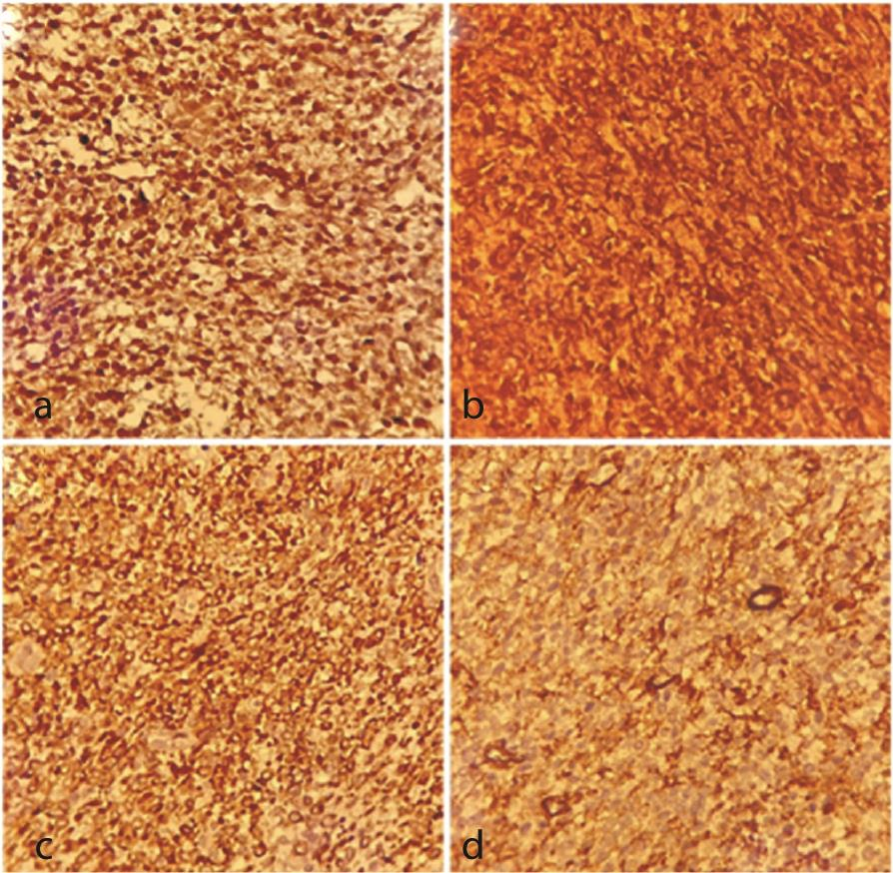
The positive markers under light microscope (x400), **a:** The tumor cell demonstrated S100 positivity, **b:** Vimentin, **c:** Desmin, and **d:** SMA

For a definite diagnosis, immunohistochemistry (IHC) staining for Ki-67 antigen, smooth muscle cell actin (SMA), and S-100 was recommended. The IHC analysis indicated diffuse and strong cytoplasmic immunostaining of tumor cells for vimentin, SMA and desmin. Patchy and weak to moderate nuclear and cytoplasmic immunostaining of S-100 was also observed in tumor cells. Positive nuclear immunostaining of Ki-67 was reported in 25-30% of tumor cells. Morphologically, low-grade spindle cell sarcoma was suspected, while the IHC analysis suggested leiomyosarcoma ( Figure 3).

### Surgical procedure

**Figure 4:**
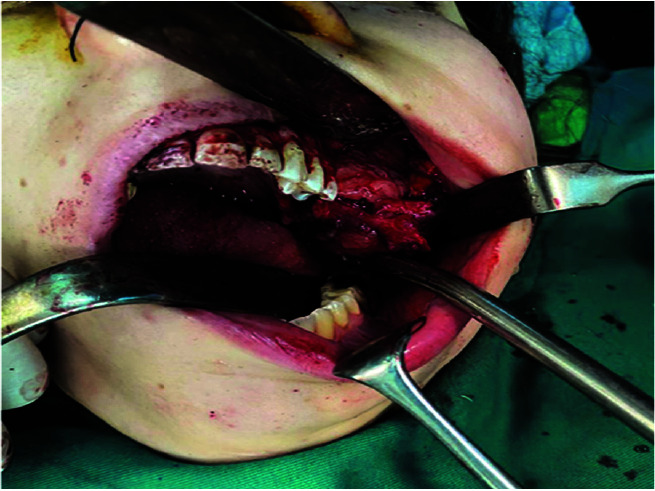
Intra oral surgical approach

Nasal intubation was performed in the operating room. Further examinations indicated the invasion of neoplastic tissue into the left maxillary sinus, causing bone erosion. Partial maxillectomy of the posterior maxillary walls was performed, using an intraoral incision and dissection from the maxillary vestibule up to the mandibular vestibule ( Figure 4). Next, the sixth tooth was extracted, and an incision was made. Finally, the tumor was excised using frozen section in which the margins were free of tumor. The specimen was sent to the pathology department for definite diagnosis. 

### Postoperative pathological exam

The results of postoperative pathology showed a malignant neoplasm, composed of a hypercellular sheet of ovoid to spindle cells with pleomorphism, hyperchromatism, and atypical mitosis. The tumor cells invaded muscle fibers, adipose tissue, perineural layers, and vessels. The pathology department reported the lesion compatible with leiomyosarcoma, with all surgical margins free of tumor.

### Second surgery

During six months after the first surgery, in regular visits, the patient complained of a gradual decreased and then very restricted mouth opening despite oral physiotherapy. First, it was diagnosed as to be due to deep scars and fibrosis in the operation site. Clinical and imaging workup showed unexpected bony fusion between the coronoid and the bone in the infratemporal area. Maximum mouth opening was decreased to less than 4-5mm when it was decided to do the second operation. Under fiber optic assisted nasal intubation and general anesthesia an uneventful intraoral coronoidectomy was performed, after which some 45 mm passive mouth opening was achieved. 

### Postoperative MRI

**Figure 5:**
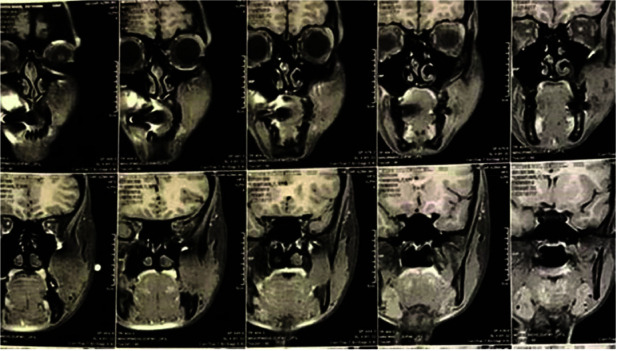
Post-operative MRI scans

Postoperative MRI scans were requested after the secocond surgery for postoperative evaluations ( Figure 5). Face and neck MRI revealed usual heterogeneously enhanced areas in the surgical bed, the left pterygoid area maxillary bone, and the masseter muscle, which could be related to postoperative changes. Moreover, mucosal thickening was observed in the left maxillary, frontal sinuses, and left ethmoid air cells. However, no definite mass was reported.

## Discussion

According to a review of the literature (shown in Table 1), leiomyosarcoma is a rare and aggressive soft tissue tumor, originating from smooth muscle cells, which tends to occur in the alimentary tract, uterus, and retroperitoneum. Only 3% of leiomyosarcoma cases occur in the head and neck regions [ 6 ]. The most common sites in the sinonasal tract include the maxillary sinus, nasal cavity and ethmoid sinuses [ 7 ]. Leiomyosarcoma of the sinonasal tract may originate from the smooth muscles of blood vessels, which are the only structures containing smooth muscles in the area [ 8 ]. Our patient was diagnosed with leiomyosarcoma in the left maxillary sinus, which is a rare observation. Generally, the treatment of leiomyosarcoma poses a challenge due to the high rate of recurrence and metastasis and poor prognosis. Our patient was 24 years old, which is the most common age for leiomyosarcoma with the age range of 21-73 years [ 9 ]. Leiomyosarcoma of soft tissue commonly presents as a slow-growing painless mass, which can cause a variety of symptoms, depending on its location [ 9 ]. Previous studies have reported leiomyosarcoma as a large soft tissue mass [ 3 , 7 , 10 - 11 ], which can invade the maxillary walls and cause obstruction and destruction [ 10 - 12 ]. The common presentations in the maxillofacial region include nasal obstruction, epistaxis, local pain, and facial swelling. Patients initially notice facial swelling with stretched skin [ 7 , 11 , 13 - 14 ]; long-term rhinorrhea is also possible [ 15 ]. In cases where the tumor invades the orbit, eye movements may be restricted [ 11 ]. The oral mucosa may remain normal or become erythematous, similar to our case, who reported oral lesions in the left maxilla five months before diagnosis [ 10 , 12 , 14 ]. Our patient had undergone a nasal polyp surgery one year before diagnosis, which could be a primary manifestation of the tumor, though without any related findings at that time. When leiomyosarcoma is suspected, a CT scan should be requested in the first step. Usually, it presents as a bulky lesion that can remodel the bone and shows mild to moderate enhancement. In addition, it commonly shows extensive necrotic or cystic changes and does not show calcification [ 16 ]. The imaging of our patient had all these features and showed mucosal thickening in the left maxillary sinus. In this regard, Taghipour *et al.* [ 6 ] reported mucosal thickening of the left maxillary sinus. In our patient, the MRI showed intermediate to high signals on the T2-weighted image, but intermediate signals on the T1-weighted image, which could be attributed to the surrounding fat and indicative of lymphadenopathy [ 10 ]. Differential diagnoses, based on all findings were a list of undifferentiated pleomorphic sarcoma, rhabdomyosarcoma, leiomyosarcoma, malignant peripheral nerve sheath tumor, fibrosarcoma, synovial sarcoma, sarcomatoid carcinoma, metastatic lesions, and fungal infections [ 6 , 14 ]. To confirm the diagnosis of the lesion, an incisional biopsy was taken. The results of the biopsy showed spindle-shaped cells with oval to elongated nuclei, mitosis, hyalinization, necrosis, eosinophilic cytoplasm, and inflammatory infiltration [ 7 , 11 , 17 ]. In our case, the IHC analysis of the specimen showed vimentin, SMA and desmin staining [ 6 , 18 ], indicative of leiomyosarcoma. The gross pathology of leiomyosarcoma indicated a firm reddish-brown appearance on the surface and a solid yellowish-white fibrous cut surface with hemorrhage and necrosis [ 5 ]; a grayish color was also observed [ 6 ]. Moreover, the pathological features were consistent with the biopsy results [ 5 - 6 ]. Studies of maxillofacial leiomyosarcoma show poor prognosis and a high recurrence rate [ 19 - 20 ]. Overall, surgical treatment with clear margins is recommended to control the recurrence of leiomyosarcoma [ 5 ]. The risk of recurrence is higher in maxillary leiomyosarcoma with a poorer prognosis, because of difficult access to some anatomical sites and free margins [ 5 , 12 ]. Overall, an accurate diagnosis and a proper combined treatment plan can produce favorable outcomes [ 5 ]. Surgery, chemotherapy, and radiotherapy are treatment modalities of leiomyosarcoma, based on clinical and tumor features. Surgical excision seems to have the best outcomes, if the tumor could be removed completely. Leiomyosarcoma is generally considered radio resistant, but the benefits of radiotherapy have been also reported. Chemotherapy is often used for metastatic lesions, as well as inoperable tumors as a palliative therapy [ 20 ]. In cases where tumor invasion is not extensive, chemotherapy may be the first-line of treatment. In this regard, Zahir *et al.* [ 6 ] used three courses of chemotherapy, including Adriamycin (35mg) plus normal saline (100cc) for three days; intravenous injection of Ifosfamide (350mg) plus  n ormal saline (500cc) for three days; and intravenous injection of Mesna (400, 800 and 800 at 0, 4, and 8 hours, respectively) for three consecutive days, along w ith intravenous injection of dacarbazine (500 mg) plus normal saline (500 cc) for three consecutive days. In addition, Nishi *et al.* [ 17 ] administered 8-10 mg/ day of Adriamycin for four days, along with cyclophosphamide. However, in their study, the patient failed to respond to treatment, and radiotherapy was administered. 

**Table 1:** Cases of maxillary leiomyosarcoma

Reference (Author) (Year)	Age (year)	Sex	Signs	Symptoms	Radiographic findings	Size	Histopathological and IHC profile	Treatment	Metastases	Outcome
Tomoki, et al. (2001)	77	M	Swelling of the left upper gingiva, second molar mobility	Swelling of the left cheek	Plain radiograph revealed a lytic lesion of the anterior hard palate and alveolar bone of the maxilla.	55×40 mm	LMS(2001)^1^, α- SMA^2+^, vimentin antibody^+^	Radiotherapy and chemotherapy	Positive	DOD^3^
Wada S, et al. (2002)	71	F	Painless mass extendin from the alveolar process of the right second premolar to the tuberosity of the maxilla	Slowly growing mass	Panoramic radiography showed resorption of the maxilla extending to the bottom of the maxillary sinus; CT showed destruction of nearly the entire tuberosity of the maxilla, with tumor invasion into the maxillary sinus	32×24 mm	LMS grade II/III	Surgery	Negative	Alive (3 years)
Rodini, et al. (2007)	63	M	A nonulcerated, nontender soft palatal mass with elastic consistency	Painless swelling of the palate	An occlusal radiograph showed homogeneous loss of trabecular architecture in the right posterior maxilla; The CT scans revealed a tumor extending into the right nasal cavity as well as into the ethmoidal region	NA	LMS, vimentin+, desmin+, α- SMA+, laminin+, HHF-35+	Surgery	Negative	NED^4^ at 12 months
Chew, et al. (2009)	36	M	A diffuse swelling, the mass filled entire nasal cavity	Painful swelling in left maxillary area	CT scans revealed a large soft tissue density in the left maxillary sinus that had destroyed all of the maxillary walls except the posterior wall	50×40 mm	LMS, vimentin+, α- SMA +	Radiotherapy	Negative	NED at 36 months
Chiu, et al. (2011)	58	M	Severe swelling over left facial area, subdermal indurated mass without skin adhesion	Swelling and pain	MRI showed a 4.8×3.5×4.7 cm sized mass lesion with partial necrosis and heterogeneous contrast enhancement, PET scan showed hypermetabolic mass located in the left maxilla with extension to level Ib cervical region	30×40 mm	LMS, α- SMA+, HHF-35+, vimentin+ and CD 146+	Surgery	Positive (6 months after surgery)	AWD^5^
Riaz, et al. (2011)	65	M	There was no palpable cervical lymphadenopathy. On intra oral examination there was a hard swelling on the palate crossing the midline. At the time of presentation, the patient was malnourished and dehydrated.	swelling	CT scans examinations demonstrated an ulcerated soft tissue density enhancing mass on the left cheek with extension into left maxillary sinus, nose, oral cavity, orbit and forehead	350×250 mm	LMS, α- SMA+	Surgery followed by adjuvant chemo/radiotherapy	Negative	NA^6^
Rahimi, et al. (2012)	36	M	A slight left paranasal swelling, oral examinations revealed a painful mass, extended from the alveolar process of the upper left second premolar to the upper right canine.	Swelling, tooth pain	The periapical radiograph of the anterior maxillary teeth revealed an ill-defined radiolucency of bone erosion and lamina dura resorption. Thus, the panoramic radiograph showed resorption of the maxilla extending up into the floor of the maxillary sinus.	35mm in diameter	LMS, vimentin+, desmin+, α- SMA +	Surgery followed by Radiotherapy	Negative	NED at 12 months
Zahir, et al. (2013)	24	M	Conjunctivitis of the right eye, a large defect in the right alveolar ridge and a mobile non-tender lymph node in the right side of neck, were detected.	Swelling, toothache, decreased vision, hemifacial paresthesia	CT scans demonstrated destructive changes of the adjacent bony structures and extension of the lesion to the soft tissue of the right masticator space, right parapharyngeal space, apex of the right orbit and skull, were also depicted.	50mm in diameter	LMS, vimentin+, α- SMA+, caldesmon	Radiotherapy and chemotherapy	NA	NED at 4 months
Papoian, et al. (2014)	83	F	A firm, smooth, and friable nasal mass along the right inferior nasal cavity	Unilateral nasal congestion, facial pain	The CT scan showed a large heterogeneously enhancing mass occupying the right nasal cavity involving the inferior turbinate and lateral nasal wall	NA	LMS, desmin+, α- SMA+	Surgery	Negative	NED at 48 months, positive history of recurrence
Sandhu, et al. (2014)	63	M	Painless and non-tender mass arose from the anterior maxillary alveolus extending from right canine to left lateral incisor	Progressive and continuous enlargement	The CT scan showed an osteolytic lesion extending in the nasal chamber but not laterally in the maxillary sinuses. The axial section showed a diffuse soft tissue mass obliterating the anterior nasal chamber completely destroying the anatomy of the anterior palate and the nasal cartilaginous skeleton	80×75 mm	LMS, vimentin+, α- SMA+, HHF-35+	Surgery	Negative	NED at 24 months
Bayramoglu, et al. (2018)	19	M	A well demarcated firm nodule extending from the left second premolar to the maxillary tuberosity without ulceration	Mobility in the teeth and limitation of function	Radiography showed a small amount of bone resorption	NA	LMS, α- SMA+	Surgery	Negative	NED at 12 months
Present case	26	F	Asymmetry of the face with swelling,	Swelling on the left side of the face	CT scan showed mucosal thickening in the left maxillary sinus, MRI revealed remodeling of the sinus wall	47×31×30 mm	LMS, vimentin+, desmin+, α- SMA +	Surgery	Negative	NED at 6 months

1.LMS: Leiomyosarcoma, 2.SMA: smooth muscle actin, 3.DOD: dead of disease, 4.NED:no evidence of disease, 5.AWD: alive with disease, 6.NA: not available

In cases where local lymph nodes are engaged, surgery with wide resection of tumor margins is recommended, given the infiltrative nature of the tumor [ 7 , 11 ]. In this regard, Pasrad *et al.* [ 11 ] used total maxillectomy, followed by radiotherapy and showed no recurrence in an 18-month follow-up. Similarly, Wada *et al.* [ 12 ] reported a three-year recurrence-free survival. However, in some cases with orbital involvement and metastasis, mortality has been reported, despite all available treatments [ 11 ]. In our case, due to the patient's emphasis on esthetics, an intraoral approach was used for tumor excision to avoid facial scarring, despite tumor invasion into the infratemporal region. Tumor excision with frozen section was carried out, the results of which were normal pathologically and were confirmed in the follow-up MRI at six months after the procedure. For treatment, eight courses of chemotherapy (Vincristine Richter 1mg/mL*2 and Endoxan®) and radiotherapy were administered. The recurrence of primary oral leiomyosarcoma is estimated at 34%, and distant metastasis has been reported to occur in 35% of patients. Lungs are the most common sites for oral leiomyosarcoma metastasis. A five-year survival rate has been reported for these patients, and metastasis involving the bone shows a poor prognosis [ 20 ]. The definitive diagnosis of leiomyosarcoma is based on histological examination, and surgical excision is the preferred method of treatment. In conclusion, according to the present results, the clinical features of leiomyosarcoma of the maxilla are clear, and diagnosis is feasible. The intraoral approach used in this case report could help avoid facial scars. However, further studies are needed to introduce a proper treatment plan for better prognosis and lower recurrence. Written and verbal informed consent for patient information and images to be published was provided by the patient.

## Conclusion

According to the present results, the clinical features of leiomyosarcoma of the maxilla are clear, and diagnosis is feasible. The intraoral approach used in this case report could help avoid facial scars. However, further studies are needed to introduce a proper treatment plan for better prognosis and lower recurrence.

## Conflict of Interest:

None declared.
